# Innovative Platform for the Advanced Online Monitoring of Three-Dimensional Cells and Tissue Cultures

**DOI:** 10.3390/cells11030412

**Published:** 2022-01-25

**Authors:** Sebastian Kreß, Roland Schaller-Ammann, Jürgen Feiel, Joachim Wegener, Joachim Priedl, Wolf Dietrich, Cornelia Kasper, Dominik Egger

**Affiliations:** 1 Department of Biotechnology, Institute of Cell and Tissue Culture Technologies, University of Natural Resources and Life Sciences, Muthgasse 18, 1190 Vienna, Austria; sebastian.kress@boku.ac.at (S.K.); cornelia.kasper@boku.ac.at (C.K.); 2 Health—Institute for Biomedicine and Health Sciences, JOANNEUM RESEARCH Forschungsgesellschaft mbH, Neue Stiftingtalstrasse 2, 8010 Graz, Austria; roland.schaller-ammann@joanneum.at (R.S.-A.); juergen.feiel@joanneum.at (J.F.); joachim.priedl@joanneum.at (J.P.); 3 Faculty of Chemistry and Pharmacy, Institute of Analytical Chemistry, Chemo- and Biosensors, University of Regensburg, Universitätsstraße 31, 93053 Regensburg, Germany; joachim.wegener@chemie.uni-regensburg.de; 4 Department of Gynecology and Obstetrics Tulln, Karl Landsteiner University of Health Sciences, Alter Ziegelweg 10, 3430 Tulln, Austria; wolf.dietrich@kl.ac.at

**Keywords:** 3D culture, perfusion bioreactor, monitoring platform, sampling, analytics, cells, microenvironment, open flow microperfusion, sensors, 3R

## Abstract

The use of 3D cell cultures has gained increasing importance in medical and pharmaceutical research. However, the analysis of the culture medium is hardly representative for the culture conditions within a 3D model which hinders the standardization of 3D cultures and translation of results. Therefore, we developed a modular monitoring platform combining a perfusion bioreactor with an integrated minimally invasive sampling system and implemented sensors that enables the online monitoring of culture parameters and medium compounds within 3D cultures. As a proof-of-concept, primary cells as well as cell lines were cultured on a collagen or gelatin methacryloyl (GelMA) hydrogel matrix, while monitoring relevant culture parameters and analytes. Comparing the interstitial fluid of the 3D models versus the corresponding culture medium, we found considerable differences in the concentrations of several analytes. These results clearly demonstrate that analyses of the culture medium only are not relevant for the development of standardized 3D culture processes. The presented bioreactor with an integrated sampling and sensor platform opens new horizons for the development, optimization, and standardization of 3D cultures. Furthermore, this technology holds the potential to reduce animal studies and improve the transferability of pharmaceutical in vitro studies by gaining more relevant results, bridging the gap towards clinical translation.

## 1. Introduction

The use of 3D cell cultures (ex vivo tissue and in vitro models) is gaining increasing importance in medicine and pharmaceutical research considering the 3R rules [1,2]. While many invasive studies have been performed on mostly live non-rodent mammalian animals historically [3], the choice of the animal model had been refined to match the more suitable model for the addressed question instead of just the available model. Hence, the variability of applied animal models has increased significantly. Many animal models share a high comparability with human genetics, anatomy, physiology, and hence, morbidity and pathology [4]. Today, by far the most used animal models are mouse and rat. Further, with selective breeding and the targeted introduction of genetic modifications, animal models have improved further to match more accurately the investigated medical phenotype. With rising awareness for animal welfare, animal rights have increased, and safety regulations defined by governmental agencies have been tightened. Today, strict regulations must be followed when planning to conduct animal experiments [5]. For the cosmetic industry, animal experiments have been forbidden already [6] as reasonable alternatives are available.

Nevertheless, for medical and pharmaceutical research and development, animal experiments are still required by regulatory agencies as cell cultures can mostly not yet reflect upon intercellular interactions or systemic effects. In particular, commonly applied 2D cultures can only complement animal data. Their results often diverge from in vivo responses, or even false positive or false negative data are generated as 2D cultures are highly artificial and thus do not represent the in vivo situation [7,8]. With the advent of in vitro established 3D cell cultures and subsequently tissue substitutes, the field of ‘tissue engineering’ emerged to create tissue models with the purpose of providing tissue grafts to reduce and eventually replace animal experiments [9]. Three-dimensional cultures replicate the in vivo microenvironment affecting migration, polarity, proliferation, and functionality by surrounding the cells with reciprocal cell–cell and cell–matrix interactions and dynamic spatial gradients of nutrients, growth factors, and stimuli. By reflecting the complexity of physiology and pathology, 3D cultures ensure more reliable and predictable results for human application. Furthermore, employing patient-derived cells enables the development of tissue models for personalized medicine [4].

Nonetheless, analytical methods have been established and optimized for 2D cell cultures. For analysis, 2D cultures have the distinct advantage of the easy availability of cells cultured in a monolayer. This facilitates the even distribution of reagents and accessibility as well as the easy retrieval of metabolites in cell-based assays. Similarly, microscopic, and spectroscopic analyses profit from the plane monolayer culture which enables clear and precise results. In contrast, analyses of 3D structures are challenging.

Microscopy is affected by the highly structured and potentially autofluorescent background of the extracellular matrix (ECM) and limited light penetration. The elevated depth of the culture requires expensive microscopes to generate sharp pictures while the penetration depth is usually lower than the tissue model, making it almost impossible to scan through the whole tissue culture [10]. To analyze 3D cultures by microscopy, the preparation of histological sections is required, which represents an endpoint measurement, making it impossible to follow the same sample over time. Furthermore, the diffusion of reagents and metabolic products in cell-based assays is hindered in 3D structures.

Moreover, the monitoring of culture conditions is similarly considerably easier in 2D cultures. In 2D cultures, the culture media supernatant is mostly representative for the cellular condition as the cells grow in a flat monolayer and thereby, they exhibit a high exchange surface. When cells are embedded in a 3D structure, the diffusion limitation is dependent on the thickness and the density of the material which establishes a gradient for nutrients and metabolites [11,12]. Thereby, the supernatant does not necessarily reflect the conditions within the matrix. However, there are no means to non-destructively sample or analyze the fluid within the 3D matrix to state an assertion on the actual culture condition. The current invasive methods cannot extract the interstitial fluid of 3D cultures, nor can sensors or analyzers determine the culture conditions reliably due to the small sample volume or the volatility of the analyte. Hence, most secretory and metabolic analysis is rather an estimation of the effective tissue condition than a reflection of the situation within the tissue.

Taken together, there is an urgent need for analytical tools and methods to monitor, optimize, and standardize the culture conditions for 3D models.

To overcome the current limitations of the non-destructive examination of 3D cultures, the aim of this study was to develop a modular cultivation and monitoring platform enabling the continuous, time-resolved online monitoring of 3D cell and tissue cultures. Herein we describe the realization and proof-of-concept demonstrating distinct discrepancies between the analysis of a medium supernatant and interstitial fluid, thus revealing the poor representation of culture conditions in 3D cultures from current standard analytical methods.

## 2. Materials and Methods

### 2.1. Rapid Prototyping

The stereolithography of bioreactor parts that are in direct contact with cells [13] were performed with the Dental SG resin (FLSGAM01, formlabs, Somerville, MA, USA) [14]. The sensor cell was printed with Clear resin (RS-F2-GPCL-04, formlabs) [15].

The polymers were printed with the SLA desktop printer Form 2 (formlabs) with an XY-resolution of 25 µm, a laser spot size of 140 µm, and a layer thickness of 25–300 µm. The dimensions of the prototype bioreactor were 16.5 × 45.5 mm. The dimension of the sensor cell was 15 × 30 × 10.5 mm. The CAD models were generated with the Solid Works^®^ (Dassault Systems SolidWorks Corp, Waltham, MA, USA) CAD package, transferred into STL files, and uploaded onto the 3D printer using PreForm software. The models were printed with a layer thickness of 50 µm since this is the best resolution that both resins were capable of (the thinnest possible layer thickness of the used resins: Clear: 25 µm, Dental SG: 50 µm).

After printing, the printed parts were washed and cured according to the manufacturer’s instructions. The support structure was removed before washing. Washing was performed in duplicate with fresh isopropanol > 98% each for 2 × 10 min for the Dental SG and for the Clear resin. After washing, the prints were allowed to air-dry. Afterwards, UV-curing was performed with a UVA-Cube 100 (Dr. Hönle AG, UV-Technologie, Gräfelfing/Munich, Germany) using a Dr. Hönle Strahler UV 150 F. The UV 150 F has a broadband spectrum from 250 nm to 600 nm with a relative intensity of 50% at 405 nm. The curing time was based on formlabs’ recommendations [16].

Dental SG were sterilized by steam sterilization for 20 min at 121 °C in an autoclave (Varioklav 500E, Thermo Scientific, Waltham, MA, USA). Clear resin was sterilized with UV-light (254 nm wavelength, 30 min each side).

### 2.2. Cell Culture

Human adipose-derived mesenchymal stem cells (adMSCs) were isolated from human tissue which was approved by the Committee for Scientific Integrity und Ethics of the Karl Landsteiner University, Austria (EK Nr: 1014/2019, 4 December 2019), and all donors gave their informed written consent. In brief, subcutaneous fatty tissue was obtained during re-laparotomies with scar resections. The scar was excised with the adherent subcutaneous fatty tissue and immediately transferred sterile in saline buffer. adMSCs were isolated within 24 h after surgery. Briefly, fat tissue was minced with scissors and digested with collagenase type I (Sigma Aldrich, St. Louis, MO, USA). Subsequently, multiple centrifugation and washing steps were carried out to receive the stromal vascular fraction, which was then transferred to cell culture flasks. MSCs were cultured in a standard medium composed of MEM alpha (Thermo Fisher Scientific, Waltham, MA, USA), 0.5% gentamycin (Lonza, Basel, Switzerland), 2.5% human platelet lysate (PL BioScience, Aachen, Germany), and 1 U/mL heparin (PL BioScience, Aachen, Germany).

Normal human dermal fibroblasts (NHDF) (PromoCell GmbH, Heidelberg, Germany) and Human Immortalized Keratinocytes (HaCaT) (DKFZ, Heidelberg, Germany) [17] were cultured in DMEM (Sigma-Aldrich, Darmstadt, Germany), 10% fetal calf serum (PAA, Pasching, Austria), and 1% penicillin/streptomycin (Gibco, Carlsbad, CA, USA).

All cell types were cultured in a humidified incubator at 37 °C and 5% CO_2_. Upon use, cells were thawed and sub-cultivated once, and were passaged at 80% confluency.

For 3D cell cultures, cells were either seeded with a density of 5 × 10^4^ cells/cm^2^ on 16 × 22 × 2 mm pieces of collagen fleece (MatriStypt, MedSkin Solutions Dr. Suwelack AG, Billerbeck, Germany) [18] or embedded in a 6% GelMA hydrogel, prepared as described by Pepelanova et al. [19,20], at a concentration of 1 × 10^6^ cells per ml and a thickness of 4 mm. The 3D cell cultures were incubated for three days in a static condition before being transferred and mounted into the monitoring platform to allow the cells to spread out in the respective ECM and adopt their typical cellular morphology after seeding into 3D.

### 2.3. Perfusion Culture

After steam sterilization of the bioreactor parts, the tubings were connected to the bioreactor to enable perfusion with a peristaltic pump. The bioreactor base was filled with the respective cell culture medium. Three-dimensional cell cultures were mounted in the mounting inserts with the open flow microperfusion (OFM) probe (PEEK002, Joanneum Research, Graz, Austria) embedded centrically within the matrices and integrated inside the bioreactor base. The cell-laden collagen fleeces were layered as a sandwich culture with the OFM probe enclosed between the cell–collagen layers. For the GelMA, the OFM probe was inserted centrically through the cell-laden hydrogel culture. After closing the bioreactor, it was kept closed at all times during the cultivation. Sensors were attached to the OFM probe for continuous online monitoring and connected to tubings (SCS001, Joanneum Research, Graz, Austria). Perfusion of the bioreactor base with culture medium was enabled by a peristaltic perfusion pump at 1 mL/min, while perfusion of the OFM probe within the 3D culture with ELO-MEL (Fresenius Kabi, Graz, Austria) was enabled with a microperfusion pump (MPP102PC, Joanneum Research, Graz, Austria) at 1 µL/min which ensures an equilibrium of the interstitial fluid from the 3D culture with the isotonic ELO-MEL solution [21]. The linear OFM probe had an open exchange area of 5 mm which allowed the unrestricted exchange of compounds via an open structure across the open exchange area at flow rates of 1 μL/min. The OFM perfusate was then monitored online with the attached sensors and subsequently collected for further offline analysis. Cells were cultivated for five days at 37 °C, 5% CO_2_, and 21% O_2_ (*n* = 3) in a customized incubator system (TERM-BioScience, Würzburg, Germany). Media supernatant and interstitial fluid perfusion samples were taken daily for subsequent offline analysis with a Cedex Bio Analyzer (Roche, Basel, Switzerland).

### 2.4. Live/Dead Cell Staining

The viability of cells was visualized with calcein AM (acetoxymethyl ester) and propidium iodide (PI) staining. Briefly, samples were stained with calcein AM (4 µM) and PI (8 µM). After washing with PBS, samples were investigated with fluorescence microscopy (Leica DM IL LED with LeicaEL6000, both Leica Microsystems GmbH, Wetzlar, Germany).

## 3. Results

### 3.1. Establishing a Modular Monitoring Platform

#### 3.1.1. Concept and Design of the Perfusion Bioreactor

In this study, we developed a perfusion bioreactor for the monitoring of 3D cell and tissue cultures. Rapid prototyping by 3D printing enabled the iteration and quick adaption of designs and enabled new and further requirements such as variations in size or geometry to be met. Peaking in the optimal shape, the final design was produced from polyether ether ketone (PEEK) (GT Labortechnik, Arnstein, Germany).

**Figure 1:**
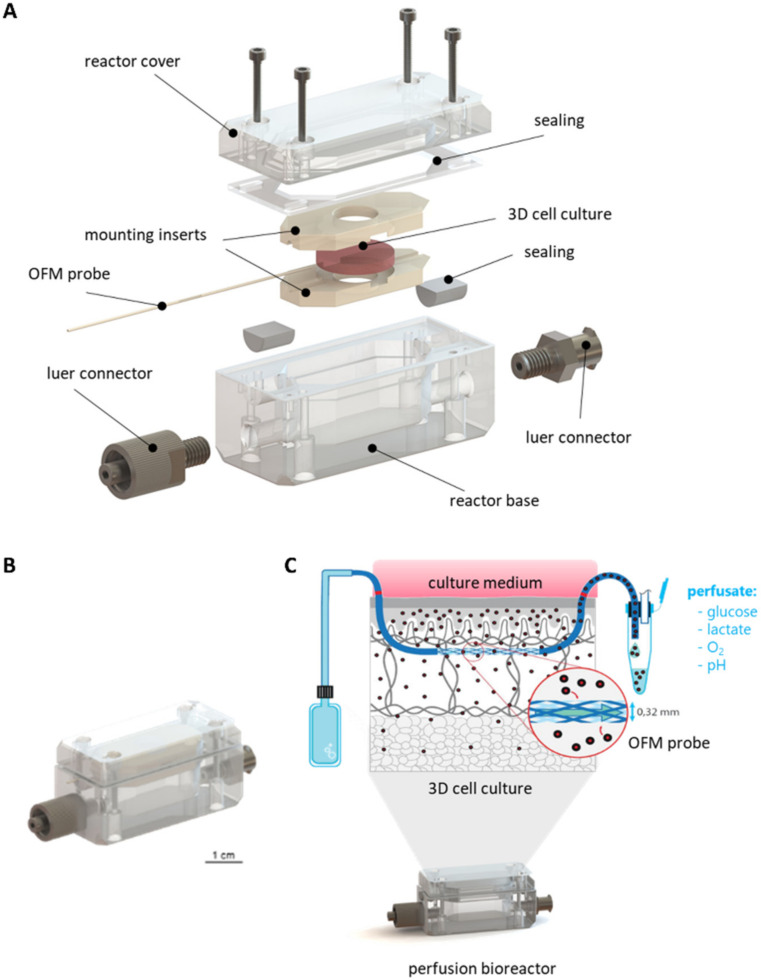
Technical description of the perfusion bioreactor system to monitor 3D cultures. (**A**): exploded view showing the individual components. (**B**): assembled bioreactor. (**C**): schematic depicting the inside of the bioreactor culture with an OFM probe integrated in a 3D cell culture. In the final setup, the bioreactor comprises a 3D cell culture with an integrated microperfusion system. The isotonic fluid in the probe equilibrates with the interstitial fluid from the 3D culture. Culture parameters, media compounds or metabolites can be analyzed via online monitoring of offline analyses.

The main body of the bioreactor consisted of one bottom chamber containing the culture medium, a mounting insert to hold the 3D cell/tissue culture, and a reactor cover closing the culture chamber (Figure 1A). To enable the connection of a tubing system for perfusion, the bioreactor was equipped with male and female luer locks (Droh, Germany) and sealed using O-rings (EPDM 3 × 1.5 mm, Shore 70, Technirub, Germany). Mounting inserts were introduced, to hold the scaffolds and the OFM probe in position. A duct ensured the placement of the OFM probe through the 3D culture as well as the bioreactor without bending the probe. Finally, the cultivation chamber, the lid, and the probe duct were sealed by a custom-made water jet cut flat gasket (Hostra, Austria) and in-house produced sealing (ELASTOSIL^®^ LR 3003/20 TR A/B—Wacker Chemie, Germany) to realize a closed system.

This setup enabled the mounting and cultivation of 3D cell and tissue cultures within a perfusion bioreactor. The integration of an OFM probe in both the bioreactor and the 3D culture enabled the sampling of the enclosed tissue rather than the supernatant (Figure 1C). Hence, the collected perfusate sample equaled the interstitial fluid in substance concentrations [21].

#### 3.1.2. Mounting of 3D Cell and Tissue Cultures

To mount 3D cultures of various sizes, shapes, and geometries, a variety of applicable mounting inserts was created (Supplementary Figure S1), enabling the system to be a platform for different kinds of tissues. Additional crosspieces also facilitated the stability of non-rigid scaffolds. All mounting inserts feature a duct for OFM probe placement.

#### 3.1.3. Development of a Sensor Platform for Online Monitoring

The integrated OFM probe within the bioreactor enabled the sampling of interstitial fluid for subsequent analysis. Implementing sensors in the modular platform facilitated immediate online analysis of culture parameters. During the proof-of-concept study, oxygen, glucose, lactate, and pH were monitored online. For implementation, sensors had to match the conditions of OFM, namely achieving reasonable repsonse times, accuracy, and precision with a flow rate of only 1 µL/min.

For glucose and lactate sensing, the afferent and efferent tubings of the Biosensor LV5 (Jobst Technologies, Freiburg im Breisgau, Germany) were exchanged for tubings with an inner diameter of 0.5 mm to reduce the dead volume of the tubings and meet the requirements of a low flow rate of 1 µL/min. Subsequently, the sensor was re-evaluated as the manufacturer’s specifications only covered flow rates down to 5 µL/min at the lowest. Preliminary experiments showed that the sensors were sufficiently stable, accurate, and precise, with an acceptable response time (data not shown). The glucose sensor displayed a dynamic range of <0.05 to 25 mM whereas the lactate sensor showed a range of <0.02 to 15 mM. Data acquisition was performed using the biosensor measuring instrumentation (Six) with incorporated temperature sensor/compensation and software (bioMON, v4.15.0), all from Jobst Technologies.

**Figure 2:**
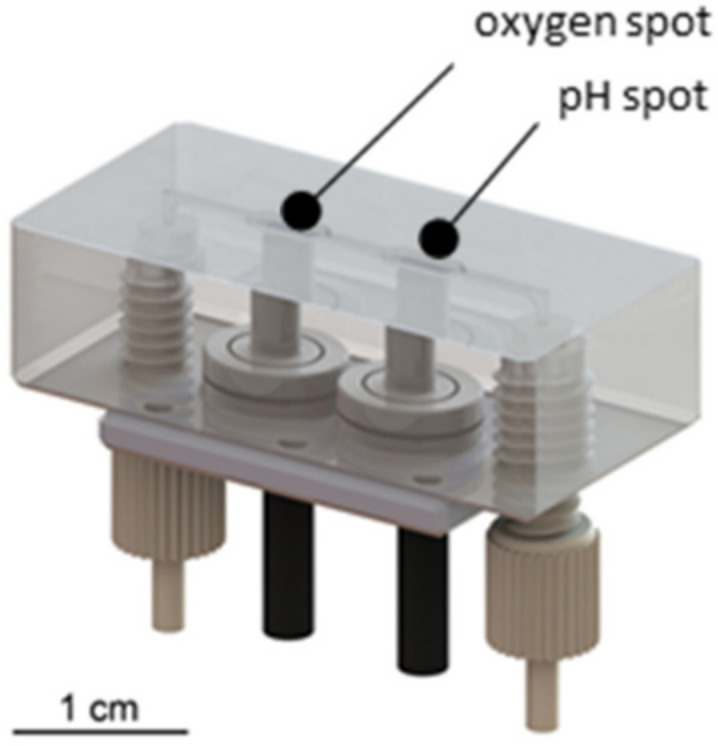
Microperfusion sensor cell with integrated optical sensor elements for oxygen and pH, optical fibres (black) and standard connectors (grey).

A prototype microperfusion flow cell for monitoring oxygen and pH provided by PyroScience (Aachen, Germany) suitable for the low perfusion speed of the OFM and featuring a minimized cell volume (~10 µL) was used. The flow cell employed optical sensor spots for oxygen and pH and was connected via optical fibres to a read-out instrument (Figure 2). The flow cell was connected to the bioreactor and the perfusate waspassed via a microperfusion channel over the oxygen and pH sensor spots. Oxygen sensors showed a dynamic range from 0 to 100% air saturation whereas pH sensors showed a range of 6 to 8 or 7 to 9, respectively. Data acquisition was performed using the optical 4 Channel pH & Oxygen & Temp Meter (FireSting^®^-PRO PyroScience).

### 3.2. Assembly of the Monitoring Platform for 3D Cell Cultures

**Figure 3:**
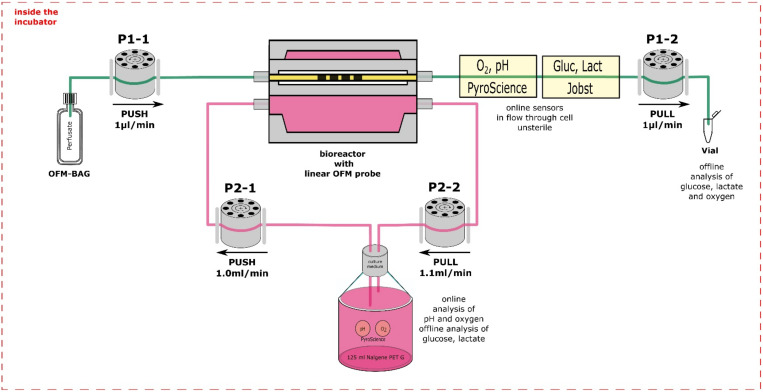
Setup of the modular monitoring platform. The bioreactor was perfused with the cell culture medium in a circuit using peristaltic pumps (P2) and the OFM probe within the bioreactor with an isotonic solution using microperfusion pumps (P1), which passed through sensors for online monitoring and was subsequently collected for subsequent offline analysis.

To assess the discrepancy of molecules, compounds and especially nutrients and metabolic waste between the culture medium and interstitial fluid, the modular monitoring platform was assembled (Figure 3). In this setup, the bioreactor with the cell-laden matrix and the OFM probe was connected to a medium perfusion circuit, operated with a peristaltic pump. The medium circuit was connected to a medium reservoir, equipped with additional sensor spots for reference online measurements of the culture parameters in the culture medium. The probe was connected to a second perfusion circuit, operated with a microperfusion pump perfusing an isotonic solution through the 3D culture. Thereby, the isotonic solution equilibrated with the interstitial fluid. This perfusate was online monitored for oxygen, pH, glucose, and lactate using two subsequent implemented flow through cells to enable monitoring of substance concentrations with temporal resolution during the sampling period of the culture.

Thereby, the online monitoring of the equilibrated perfusate in the probe, and the culture medium in the medium reservoir, allowed the comparison of the bulk media supernatant and the interstitial fluid from the 3D cultures. Furthermore, perfusate samples were collected frequently in attached vials for subsequent bioanalytical offline analysis. All equipment was placed inside the incubator.

### 3.3. Discrepancy between Media Supernatant and Interstitial Fluid

To demonstrate the discrepancy of molecules, compounds, and especially nutrients as well as metabolic waste between culture medium and interstitial fluid, cell-laden scaffolds were cultured within the established monitoring platform while the media supernatant and interstitial fluid were directly compared by online and offline monitoring. To do so, the monitoring platform was assembled as described above. Online monitoring with temporal resolution of critical culture parameters inside of 3D cell cultures was performed: oxygen, pH, glucose, and lactate concentration within both, the 3D culture interstitial fluid extracted by the OFM compared to the respective concentrations within the media supernatant. Subsequently, concentrations of glutamate, glutamine, and ammonia in the interstitial fluid and media supernatant were measured offline. At the end of the 5-day culture, the viability of cells was analyzed via live-dead staining of the cell-laden matrices.

For this proof-of-concept study, primary cells, and cell lines in combination with a collagen fleece or GelMA were used as follows: (i) normal human dermal fibroblasts on collagen fleece, (ii) HaCat keratinocytes on collagen fleece, (iii) adMSCs on collagen fleece, and (iv) adMSCs embedded in a 6% GelMA hydrogel.

#### 3.3.1. Three-Dimensional Culture of Fibroblasts and Keratinocytes

**Figure 4:**
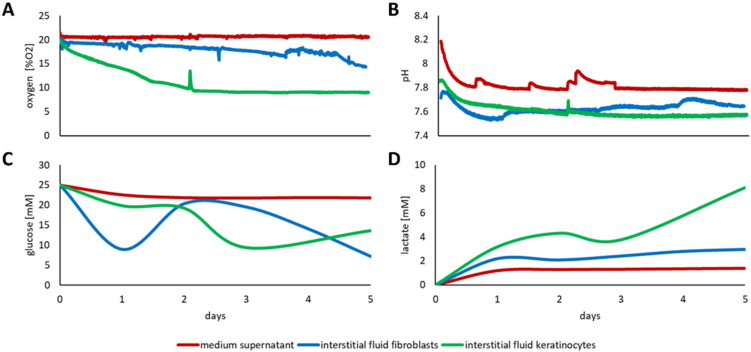
Discrepancy of the effectively present culture conditions between the medium supernatant and the interstitial fluid of fibroblasts and keratinocytes in a collagen fleece. Online monitoring within the monitoring platform of (**A**) oxygen, (**B**) pH, (**C**) glucose, and (**D**) lactate concentrations in the medium supernatant (red line,) and the interstitial fluid for fibroblasts (blue line) and keratinocytes (green line).

The online monitoring of fibroblasts and keratinocytes demonstrated considerable discrepancies between concentrations, determined within the cell culture supernatant when compared to the respective concentrations within the interstitial fluid of the 3D cell cultures. While monitoring the culture conditions for the fibroblasts, within the 3D scaffold slightly below normoxic culture conditions were determined, the oxygen saturation within the keratinocyte populated 3D scaffold dropped to 10% oxygen saturation, half of which was measured within the media supernatant (Figure 4A). The difference in pH between media supernatant and interstitial fluid was about 0.2–0.3 for both, fibroblasts, and keratinocytes, with the values being steady throughout the experimental course (Figure 4B).

Examining glucose concentrations, a substantial difference between the culture medium and the interstitial fluid of the 3D culture was observed for both cell types (Figure 4C). While the glucose level in the medium remained constant, the glucose concentration in the fibroblast seeded scaffold fluctuated remarkably. During the first day of culture, there was a substantial decrease in the glucose detected to almost a third of the glucose available in the supernatant, which was then recovered, presumably by media exchange due to the perfusion culture, but then steadily declined over time. For keratinocytes, the glucose concentration decreased only slightly below the medium glucose concentration during the first days within the media. Subsequently, the glucose concentration in the 3D culture decreased to half of the glucose concentration available within the medium. Moreover, the lactate concentration substantially increased during the culture period for both, fibroblasts and keratinocytes, which was not reflected in the media supernatant (Figure 4D). The lactate concentration in the fibroblast-seeded 3D culture accumulated consistently to more than twice the concentration of the detected concentration within the supernatant. After two days of the keratinocyte culture, the lactate concentration exceeded the concentration in the supernatant more than threefold, rising to above fivefold by end of the culture.

Besides online monitoring with integrated sensors, the perfusate and supernatant were continuously sampled and subsequently analyzed for glutamine, glutamate, and ammonia. While the glutamine concentration did not differ essentially between the medium supernatant and interstitial fluid from fibroblast culture (Supplementary Figure S2A), there was up to ten times more glutamate available in the supernatant compared to the 3D culture (Supplementary Figure S2B). For HaCaT, there was no glutamine nor glutamate detectable in the sample’s interstitial fluid. Furthermore, ammonia concentrations between supernatant and fibroblast interstitial fluid did not differ substantially, while up to double the concentration accumulated in the keratinocyte interstitial fluid (Supplementary Figure S2C). Finally, the live-dead staining of the 3D fibroblast and keratinocyte cultures revealed viable cells in both conditions, proving the bioreactor to be a suitable cultivation system for 3D culture for both cell types (Supplementary Figure S2D,E).

#### 3.3.2. Three-Dimensional Culture of Primary adMSCs

In addition to fibroblasts and keratinocytes, primary MSCs were also cultured and monitored. MSCs were also seeded on a collagen matrix and embedded into a GelMA hydrogel. Again, the online monitoring of oxygen, pH, glucose, and lactate revealed remarkable discrepancies between the media supernatant and the interstitial fluid in the 3D cell cultures.

**Figure 5:**
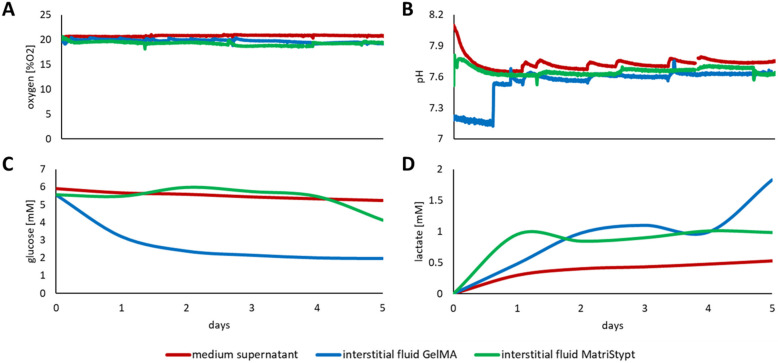
Discrepancy of the effectively present culture conditions between the medium supernatant and the interstitial fluid of MSCs in GelMA and in a collagen fleece. Online monitoring within the monitoring platform of (**A**) oxygen, (**B**) pH, (**C**) glucose, and (**D**) lactate concentrations in the medium supernatant (red line,) and the interstitial fluid from GelMA (blue line) and MatriStypt (green line) 3D culture.

While the difference of oxygen and pH between the media supernatant and the interstitial fluid was not significant in the 3D MSC cultures (Figure 5A,B, respectively), there was a vast divergence in available glucose when comparing the media supernatant with the interstitial fluid of the GelMA culture (Figure 5C). While the overall glucose concentration over the course of the culture was neglectable, the available glucose concentration in the interstitial fluid was less than half of the medium concentration after one day of culture. Lactate analyses of media supernatant and interstitial fluid of the MSC GelMA and MatriStypt culture also differed. The accumulated concentration of lactate was almost consistently more than two to three times higher in the 3D culture than in the supernatant (Figure 5D). Additional offline monitoring of glutamate, glutamine, and ammonia revealed that the culture conditions in the culture supernatants and interstitial fluids of the 3D cultures differed by about 20% (Supplementary Figure S3A–C, respectively). However, within the first day of culture there was an initial substantial reduction to half of the available glutamine and glutamate in the MSC GelMA culture compared to the respective availability in the supernatant. To confirm the viability of the cells after the culture, the cells were positively stained with calcein AM in the matrices (Supplementary Figure S3D,E).

## 4. Discussion

Sophisticated systems for continuous, time resolved, non-destructive analysis of 3D models have not been available so far, which has limited the full exploitation of 3D cultures for basic and pharmaceutical research. There are probes available to be mechanically inserted into 3D cultures but monitoring only one analyte each. Available non-invasive methods only have a penetration depth of about 1 mm and therefore cannot deliver representative information on the conditions inside of relevant 3D cultures. Further, endpoint analyses which are often required to investigate 3D cultures do not allow the same 3D model to be studied over time. This would allow a reduction in sample size, costs and variations. Consequently, well-defined, standardized and physiologically relevant human 3D cultures have the potential to replace animal models and yield relevant data for translation to clinical applications.

In this study, we established an innovative platform for cultivation and online monitoring of 3D cell and tissue cultures. Integrating OFM, a proven probe-based in vivo sampling method [21,22] that is used to evaluate the pharmacokinetics and pharmacodynamics of drugs directly in skin [23], adipose [24], and brain [25] tissue in vivo, in a bioreactor opens new horizons for its applicability in in vitro models and tissues. The further implementation of sensors to the OFM in the bioreactor complements the monitoring platform enabling the sampling and online monitoring of the interstitial fluid. Thereby, cellular processes, such as developmental, differentiation, inflammation, or healing, can be monitored with temporal resolution. The gained insights can be utilized for the optimization of tissue models and their culture conditions or to improve the transferability of in vitro studies towards the in vivo situation.

The proof-of-concept for the monitoring platform was performed with 3D cultures of fibroblasts, keratinocytes, and mesenchymal stem/stromal cells (MSCs) in collagen and gelatin-methacryloyl (GelMA) matrices. Fibroblasts and keratinocytes depict the most common cell types and collagen, which is the most abundant ECM protein in the skin [26], for which they are the most developed and used tissue models in commercial application [27]. Furthermore, primary human MSCs depict one of the most investigated cell types for cell therapy applications [28,29,30] due to their differentiation potential [31] as well as their applicability for wound healing [32], angiogenesis [32,33] and immunomodulatory properties [34,35,36]. Moreover, GelMA hydrogels represent an emerging versatile matrix for 3D cell culture [19,37]. Finally, we monitored the most universal and crucial cell culture parameters with oxygen, pH, glucose, and lactate as proof-of-concept for the developed monitoring platform. Oxygen is crucial for cellular respiration. Nevertheless, for most tissues, the physiological oxygen level is below the atmospheric oxygen level for the cells to maintain tissue specific characteristics and functionality [38,39]. Furthermore, hypoxic oxygen concentration is critical for tumor progression and malignancy of cancer cells in 3D models [40,41]. With reduced oxygen availability, cells switch to anaerobic respiration increasing lactate production and secretion and thereby acidification of the extracellular environment [42]. This is also a trait in cancer cells, which usually exhibit a pH of 6 to 7. In addition, the pH is also an important factor during wound healing [43]. Lactate, on the one hand, is a metabolic waste product of hypoxia-induced glycolysis and a reason for acidosis-induced tissue damage; on the other hand, it is a regulatory metabolite for intercellular communication, e.g., during wound repair [44]. Glucose is the major cellular carbon source and therefore significantly impacts cellular metabolism and, hence, most cellular processes [45]. Glucose metabolism is enhanced in cancer cells due to increased anaerobic glycolysis [46].

Embedding the OFM probe in in vitro tissue models inside the perfusion bioreactor and implementing sensors allowed the time resolved online monitoring and sampling of interstitial fluid from within the 3D cultures. Our results clearly demonstrate that the analysis of the undiluted interstitial fluid is more representative than the current gold standard, the analysis of the media supernatant. For example, during the culture of keratinocytes on a collagen matrix, the ambient oxygen concentration was set to 21% which was also measured in the medium. However, a hypoxic environment (about 10% O_2_) developed in the 3D culture, along with considerably lower glucose concentrations and the accumulation of lactate. Although oxygen, pH, glucose and lactate can fundamentally impact cellular behavior [47,48], these parameters have hardly been monitored in 3D cultures so far, resulting in low reproducibility and standardization of 3D culture processes. Furthermore, the absence of detectable glutamine and glutamate in the interstitial fluid of the keratinocyte culture might indicate the high uptake rate of HaCaT cells compared to the diffusion rate from the media supernatant into 3D tissue to not allow glutamate or glutamine accumulation. Indeed, our study demonstrates that it is insufficient to monitor basic culture parameters in the culture medium to fully control 3D culture processes. Thus, demonstrating the power of the platform to indicate the actual culture conditions in temporal resolution while monitoring the supernatant might provide false indications on cellular viability.

The combination of OFM sampling and a perfusion bioreactor enables the long-term cultivation of in vitro 3D cell and tissue cultures as well as the continuous non-destructive time resolved analysis of the culture conditions inside the culture model. Furthermore, the implementation of sensors facilitates the real time monitoring of culture parameters in the 3D culture. This allows for immediate time resolved recognition of changes of metabolic compounds, tissue viability and functionality, dependent on the respective sensors. The time resolved recognition of changes in culture conditions is especially important for developing tissue models with a strict timely orchestrated differentiation process. If the metabolites in the supernatant are only measured while being unaware of a time delay of their availability due to diffusion limitations caused by the ECM the introduced correction measures will not solve the cause but only treat the symptom. We demonstrated that depending on the cells and the ECM used, the difference between the analytes present in the supernatant and the interstitial fluid can be tremendous. Thus, when optimizing the culture conditions, the supernatant analysis will not reveal the actual condition inside the tissue model. Hence, necessary optimization steps might remain unnoticed or could be implemented excessively. Moreover, with ubiquitous culture conditions such as oxygen and glucose availability, which are vital for general cellular behavior and viability [49], demonstrating a vast difference, the impact might be even more pronounced for specific parameters for development, differentiation, inflammation, or wound healing. Besides specific tissue models and applications, the monitoring platform can be utilized to improve general culture conditions of 3D cell constructs investigating the availability of oxygen, pH, glucose, lactate, but also of amino acids, lipids, growth factors, trace elements and serum, or platelet lysates [50].

Finally, the monitoring platform revealed distinct differences between both fluids and demonstrated that the media supernatant in a perfusion bioreactor setup is not representative for the culture conditions within the 3D culture. Hence, the conclusions drawn from media supernatant analyses might lead to wrong assumptions on the actual conditions for 3D cell and tissue cultures. This discrepancy might be even more pronounced in static culture conditions where active mass transfer is not supported without perfusion. Thus, the supernatant is optimized to optimal culture conditions neglecting the immediate conditions within a dense 3D matrix with diffusion limitations impeding the supply and replenishment of consumed nutrients resulting in a depletion as well as the accumulation of waste and by-products. Otherwise, the OFM-supported culture monitoring enables the depiction of the actual conditions being able to resolve changes in culture parameters inside the tissue. Accurate information about the culture conditions could also facilitate the optimization of feeding strategies, thus improving (long-term) the cultivation and advancement of 3D cell and tissue culture models as well as their significance. Furthermore, a time resolved continuous analysis reveals kinetic effects and sequential processes which might stay hidden when investigated by single end point analyses. Consequently, more physiologically representative tissue models can be generated yielding more in vivo representative results bridging the gap towards clinical translation.

For future studies, we aim to use this platform to establish physiological culture conditions for standardized maturation or differentiation processes as tissue engineered 3D models (i.e., full skin equivalents) play a major role in pharmaceutical testing.

Furthermore, modifications of the bioreactor will allow the penetration and distribution of applied drugs within the tissue to be studied. As even small changes in the secretory behavior of cells become assessable, the presented system will improve the validity and relevance of such studies. Moreover, the embedded probe might also be used to inject and distribute a substance of interest directly inside the 3D tissue, rather than applying it via the medium circuit.

## 5. Conclusions

The herein presented modular monitoring platform represents a novel platform technology for the continuous minimally invasive monitoring of 3D cultures. The direct access to the interstitial fluid of 3D cultures enables continuous time resolved sampling and online monitoring in a non-destructive manner. It facilitates a more detailed insight into the conditions and processes within 3D cell cultures and tissue models empowering the user to optimize 3D cultures and improve the in vivo transferability. These results underline the importance of digging deeper inside the tissue for an analysis of the actual culture conditions instead of scratching the surface by examining the supernatant, eventually concluding from not representative results. The presented perfusion bioreactor for 3D cultures in combination with the microfluidic sensor platform opens new horizons for the development, optimization and standardization of 3D tissue models and 3D culture processes. As a consequence, this technology holds the potential to reduce animal studies and improve the transferability of pharmaceutical in vitro studies by gaining more relevant results, bridging the gap towards clinical translation.

## Data Availability

The data presented in this study are available on request from the corresponding author.

## Supplementary Materials

The following are available online at https://www.mdpi.com/article/10.3390/cells11030412/s1, Figure S1: Mounting inserts for 3D cell and tissue cultures, Figure S2: Discrepancy of effectively present culture conditions between medium supernatant and interstitial fluid of fibroblasts and keratinocytes in a collagen fleece, Figure S3: Discrepancy of effectively present culture conditions between medium supernatant and interstitial fluid of MSCs in GelMA and in a collagen fleece.
