# Supplementary material for: Innovative Platform for the Advanced Online Monitoring of Three-Dimensional Cells and Tissue Cultures
Source: Cells. 2022 Jan 25;11(3):412. doi: 10.3390/cells11030412 (PMC8834321; doi:10.3390/cells11030412)
Supplement: Supplementary file 1 [file cells-11-00412-s001.zip › cells-1533309-supplementary.pdf]

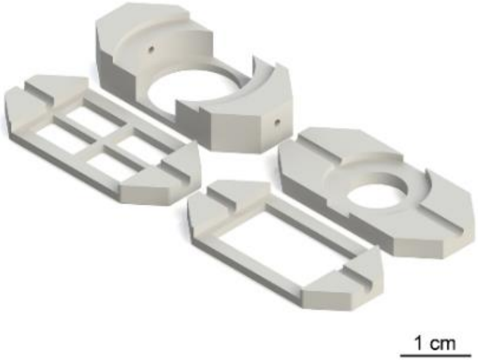

Supplementary Figure S1: Mounting inserts for 3D cell and tissue cultures.

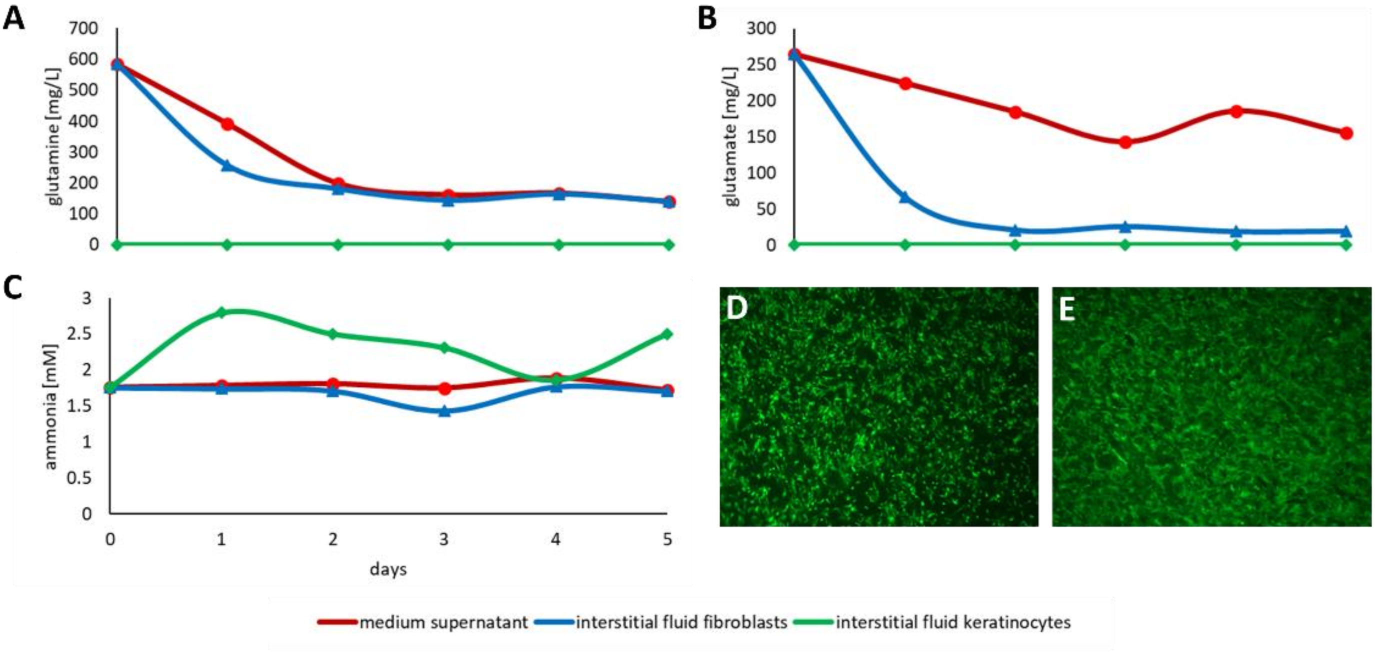

Supplementary Figure S2: Discrepancy of effectively present culture conditions between medium supernatant and interstitial fluid of fibroblasts and keratinocytes in a collagen fleece. Offline monitoring within the monitoring platform of (A) glutamine, (B) glutamate, and (C) ammonia in medium supernatant (red line,) and interstitial fluid for fibroblasts (blue line) and keratinocytes (green line). Viability staining with calcein AM (green, live) and PI (red, dead) staining of (D) fibroblast and (E) keratinocyte seeded collagen matrix after culture. Standard deviation not shown due to clarity of presentation (*n* = 3). Scalebar 100 µm.

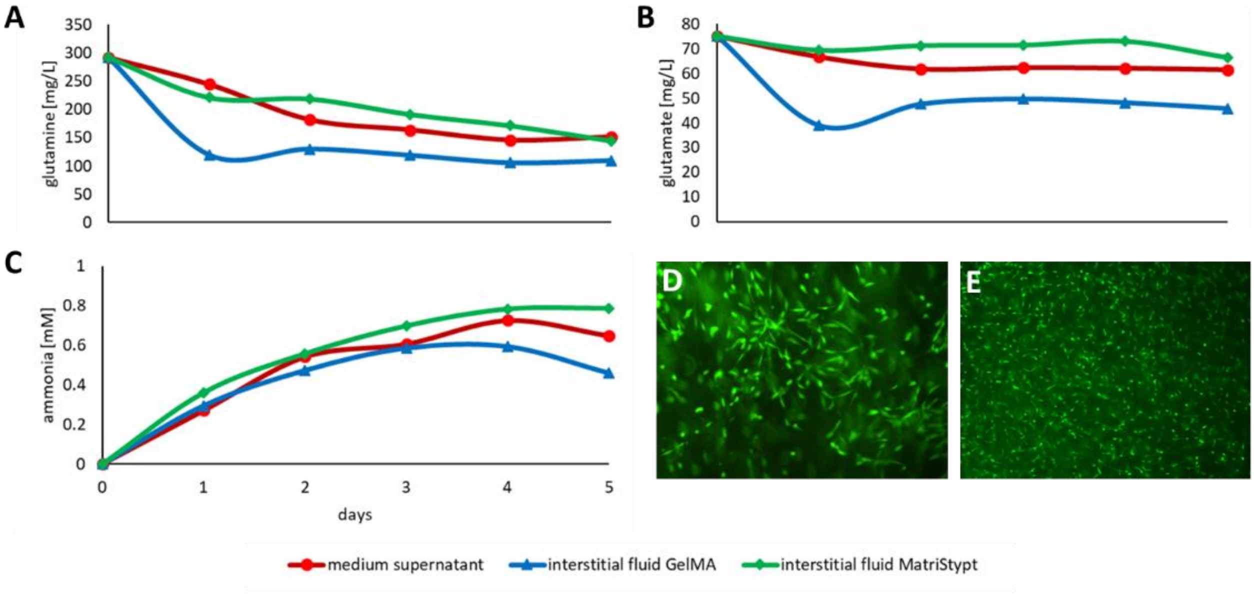

Supplementary Figure S3: Discrepancy of effectively present culture conditions between medium supernatant and interstitial fluid of MSCs in GelMA and in a collagen fleece. Offline monitoring within the monitoring platform of (A) glutamine, (B) glutamate, and (C) ammonia, for medium supernatant (red line,) and interstitial fluid from GelMA (blue line) and MatriStypt (green line) 3D culture. Viability staining with calcein AM (green, live) and PI (red, dead) staining of (D) GelMA and (E) MatriStypt seeded MSC cultures. Standard deviation not shown due to clarity of presentation (n=3). Scalebars 100 µm.
